# Evaluation of Virtual Touch Tissue Imaging Quantification, a New Shear Wave Velocity Imaging Method, for Breast Lesion Assessment by Ultrasound

**DOI:** 10.1155/2014/960262

**Published:** 2014-03-31

**Authors:** Michael Golatta, Mirjam Schweitzer-Martin, Aba Harcos, Sarah Schott, Christina Gomez, Anne Stieber, Geraldine Rauch, Christoph Domschke, Joachim Rom, Florian Schütz, Christof Sohn, Jörg Heil

**Affiliations:** ^1^Breast Unit, University of Heidelberg, Im Neuenheimer Feld 440, 69120 Heidelberg, Germany; ^2^Institute of Medical Biometry and Informatics, University of Heidelberg, 69120 Heidelberg, Germany

## Abstract

*Objectives*. To evaluate virtual touch tissue imaging quantification (VTIQ) as a new elastography method concerning its intra- and interexaminer reliability and its ability to differentiate benign from malignant breast lesions in comparison to and in combination with ultrasound (US) B-mode breast imaging reporting and data system (BI-RADS) assessment. *Materials and Methods*. US and VTIQ were performed by two examiners in 103 women with 104 lesions. Intra- and interexaminer reliability of VTIQ was assessed. The area under the receiver operating curve (AUC), sensitivity, specificity, positive predictive value (PPV), and negative predictive value (NPV) of BIRADS, VTIQ, and combined data were compared. *Results*. Fifty-four of 104 lesions were malignant. Intraexaminer reliability was consistent, and interexaminer agreement showed a strong positive correlation (*r* = 0.93). The mean VTIQ values in malignant lesions were significantly higher than those in benign (7.73 m/s ± 1.02 versus 4.46 m/s ± 1.87; *P* < 0.0001). The combination of US-BIRADS with the optimal cut-off for clinical decision making of 5.18 m/s yielded a sensitivity of 98%, specificity of 82%, PPV of 86%, and NPV of 98%. The combination of BIRADS and VTIQ led to improved test validity. *Conclusion*. VTIQ is highly reliable and reproducible. There is a significant difference regarding the mean maximum velocity of benign and malignant lesions. Adding VTIQ to BIRADS assessment improves the specificity.

## 1. Introduction

Breast ultrasound (US) is used to differentiate between benign and malignant lesions. The B-mode technique is commonly applied to evaluate morphologic features as, for example, shape and margin of a lesion [1–4]. Unfortunately B-mode US suffers from varying specificity [5–7].

A newer development is elastography, which can measure the stiffness of tissue [8, 9]. Within the last few years various studies have evaluated different methods of elastography showing that elastography has the same diagnostic performance as B-mode US and in combination with B-mode US it can help improve the differentiation of benign and malignant lesions [10–12]. Since malignant lesions often alter tissue stiffness the basic hypothesis of all elastography techniques is that malignant tissue is stiffer than benign tissue. This can help to differentiate them [13–16].

First studies on strain elastography showed several disadvantages; most meaningful is the interexaminer variability concerning the acquisition and interpretation and the qualitative strain information [17, 18]. In spite of these limitations of the method the diagnostic accuracy of elastography was at least as good as that of standard B-mode US breast imaging reporting and data system (BI-RADS) assessment, which provides standardized terminology to describe US mass features, assessments, and recommendations [19–22]. In order to improve the diagnostic quality in terms of sensitivity, specificity, and predictive value examiner independency and reproducibility have to be addressed. One possibility to improve the diagnostic accuracy of elastography would be a highly reproducible quantitative method which could be compared objectively. The virtual touch tissue imaging quantification (VTIQ) method has the potential to technically overcome the limitations of former approaches of strain elastography because it is meant to be less examiner dependent and reproducible and results in an absolute measurement of tissue stiffness in the region of interest.

The aim of this study is to prospectively evaluate this new method of virtual touch tissue imaging quantification (VTIQ) concerning its intra- and interexaminer reliability. In addition its ability of differentiating benign from malignant lesions on the basis of the lesion's stiffness will be evaluated and compared to conventional B-mode US BI-RADS assessment and the combination of both methods.

## 2. Material and Methods

### 2.1. Virtual Touch Tissue Imaging Quantification (VTIQ)

Virtual touch tissue imaging quantification (SIEMENS Medical Solutions, Mountain View, CA, USA) is a new elastography technique. Early elastography relied on manual compression and decompression applied by the examiner [23]. To improve examiner independency and reproducibility the tissue compression is automated in VTIQ. The probe generates a longitudinal push pulse which causes minimal localized displacement and is tracked by a detection pulse [12, 24, 25]. Therefore—compared to other available elastography techniques—measuring the shear wave propagation induced by the automated push pulse is meant to be the most standardized and examiner independent technique [18]. For VTIQ we used a 9 MHz probe (9L4 Siemens) equipped with the ability of generating a low frequent longitudinal push pulse. The push pulse induces shear waves which travel perpendicular to the ultrasound beam [26]. VTIQ measures the speed of the perpendicular shear waves by detection pulses. Because the speed of the shear waves propagating through the tissue is proportional to the stiffness of the tissue, a color coded map in the region of interest (ROI) gives information on the tissue stiffness [18, 26]. The shear wave velocity can be quantitatively measured in meters per second (m/s) within the ROI, up to 8.40 m/s [24]. Manual precompression of the tissue changes the elasticity and makes the tissue stiffer [27]; to obtain the most reproducible and optimal images it is therefore essential to minimize the precompression. Because shear waves cannot propagate in vicious fluid, no signal can be measured, for example, in cysts [28, 29].

### 2.2. Patients and Study Design

The patients involved in the study were referred to a specialized diagnostic breast clinic to clarify clinical symptoms or already assumed imaging abnormalities. Before scanning with VTIQ the women underwent standard clinical routine imaging. This work-up consisted of a clinical examination, ultrasound, and mammography if clinically indicated. BI-RADS assessment was completed after the routine work-up by the consultant and before VTIQ was applied. VTIQ is an absolute measurement which cannot be influenced by the prior BI-RADS assessment; therefore it can be performed by the same examiner without the risk of a bias.

After being examined women of the age of 18 or older with focal breast lesions assessed BI-RADS 3 to 5 visible in standard ultrasound were invited to participate in the study. From May to August 2012 a total of 125 patients were screened for the study. Twenty-two patients refused consent to participate in general or to be biopsied. In total 103 patients with 104 lesions gave informed consent and were finally examined using VTIQ.

One hundred lesions categorized BI-RADS 3, 4a, 4b, 4c, or 5 underwent ultrasound guided biopsy (BIP HistoCore 14 G). In these cases histology was used as gold standard. Four cysts categorized BI-RADS 3 were aspirated. In these cases the clinical diagnosis was used as gold standard. For this study biopsy was an inclusion criterion. All patients were scanned with the ultrasound system ACUSON S3000 US unit equipped with the VTIQ software (SIEMENS Medical Solutions, Mountain View, CA, USA). Each patient was scanned by a consultant specialized in breast imaging and an inexperienced examiner (beginner). Both examiners had been trained by a SIEMENS sonographer. Prior the study recruitment 25 patients were scanned with the VTIQ which were not included in the study to avoid influencing the study results by inexperience.

Step by step the consultant selected the lesion with the 9 MHz probe at its largest dimension in B-mode and adjusted the size of the VTIQ measuring box to include the lesion and surrounding tissue. For VTIQ examiner 1 (consultant) measured the maximum velocity within the lesion by selecting the ROI. In some cases the lesions showed a surrounding high velocity ring and in these cases the measurement was taken within the ring. The velocity measurement of the lesion was repeated five times. The second set of five VTIQ images was then obtained by examiner 2 (beginner) in the same way to test the reliability and the examiner independency of VTIQ. We intentionally chose an inexperienced examiner 2 to assess the influence of experience in breast US for VTIQ.

### 2.3. Statistical Analysis

This study is of explorative character. All statistical analyses are of descriptive nature. Statistical tests and resulting *P* values are not adjusted for multiplicity and can therefore only be interpreted descriptively. To begin with, the study cohort was described by the measures of the empirical distribution. Depending on the scale level of the variable, mean and standard deviation, median and quartiles, minimum and maximum, and absolute and relative frequencies are calculated. In a second step, the intra- and interexaminer reliability was assessed. To calculate the intraexaminer reliability, the VTIQ values of the five measurements of each examiner were compared by calculating the range of these values (minimal value–maximal value). A range of zero thus corresponds to perfect agreement whereas a range larger than zero reveals that the five measurements deviate. The mean range over all patients and corresponding standard deviation was calculated for both examiners. In order to assess interexaminer reliability, the means of the five measurements were compared between examiners 1 and 2. Pearson correlation coefficient was calculated and an orthogonal regression line was fitted. A slope close to 1 and a constant close to zero would thus correspond to good agreement between raters.

In order to assess diagnostic accuracy for VTIQ and BI-RADS, an ROC analysis was performed for each of the diagnostic instruments. The optimal cut-off was determined by considering sensitivity and specificity but focusing on sensitivity due to its higher clinical relevance. Moreover positive and negative predictive values were calculated. These statistical measures of the test performance are specific for this study population and can only be used to compare the different methods (BI-RADS, VTIQ, and the combining of VTIQ and BI-RADS). Particularly specificity cannot be compared to other studies because no BI-RADS 2 and only a few BI-RADS 3 cases were included in our study contrarily to other similar studies (see Sections 3 and 4). To assess the improvement of diagnostic accuracy by combining VTIQ and BI-RADS a logistic regression was performed. Using the logistic regression model, positive predictive values can be calculated for each combination of VTIQ and BI-RADS categories. The positive predictive values can thus be interpreted as a function of VTIQ and BI-RADS yielding a transformation of the two variables into a one-dimensional space. An extended ROC analysis based on the positive predictive function can thus be performed in order to directly compare the improvement of the combination of VTIQ and BI-RADS to BI-RADS alone.

All analyses were done using software SAS JMP version 6.0.

## 3. Results

### 3.1. Description of the Study Cohort

The final analysis was based on 104 lesions in 103 patients (mean age 51 years ± 18.56, range 20–89 years). The mean imaging size of the palpable lesions was 21 mm ± 10.06 (range 7–53 mm) and of the nonpalpable 13 mm ± 5.98 (range 6–32 mm). Histological examination showed 50 benign (48.1%) with a mean size of 18.1 mm ± 10.4 (range 6–53 mm) and 54 malignant lesions (51.9%) with a mean size of 17.7 mm ± 8.7 (range 7–38 mm).  Table 1 lists the absolute and relative frequencies of each BI-RADS category, the mean and standard deviation for the VTIQ values for both examiners and the histologic diagnoses of the examined benign and malignant breast lesions.

### 3.2. Intra- and Interexaminer Reliability

For each lesion, the range (maximum minus minimum) of the five measurements was calculated for examiner 1 (consultant) and for examiner 2 (beginner). For examiner 1, the mean range was given by 1.05 m/s ± 0.85, indicating that the intraexaminer measurement agreement deviates on average about one score unit while examiner 2 had a mean range of 0.92 m/s ± 0.74 which corresponds with a slightly better agreement.

In order to assess the interexaminer reliability, an orthogonal regression between the mean measurements of the two examiners was performed. The resulting regression line has a slope of 1.07 and a constant of −0.91 indicating that the agreement was good but that the rating of examiner 2 was systematically lower than that for examiner 1. Pearsons correlation coefficient was given by *r* = 0.93.

### 3.3. Differentiation of Benign and Malignant Lesions

**Figure 1:**
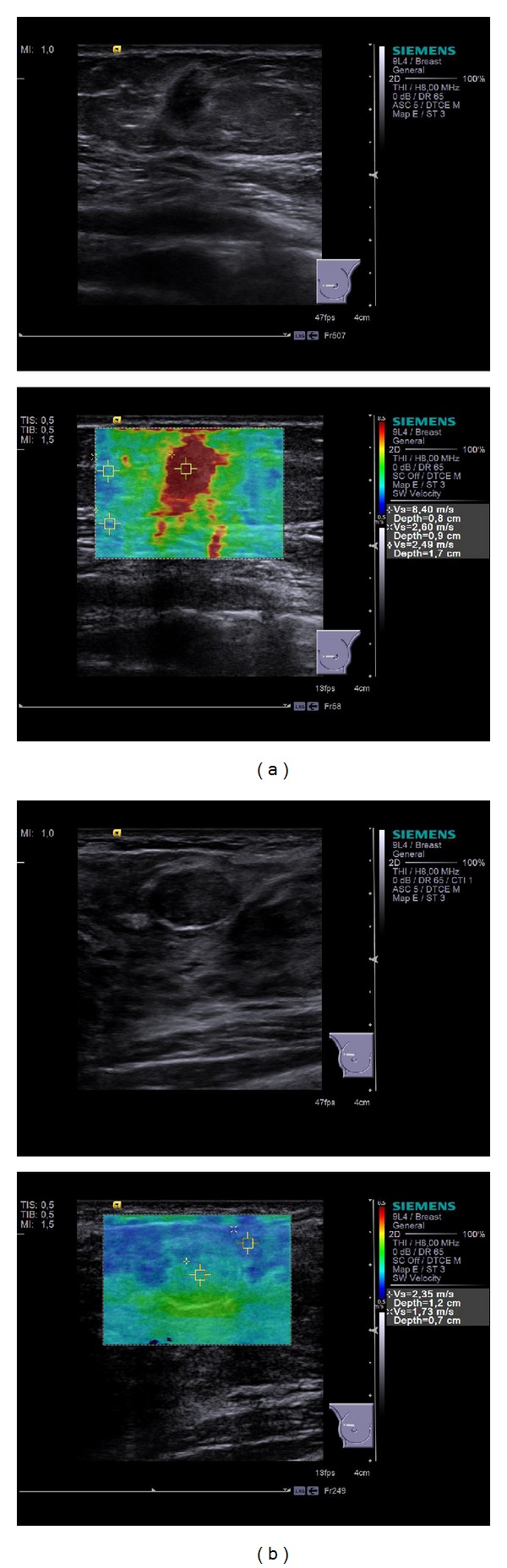
(a) B-mode and VTIQ images of a 55-year-old woman show an invasive lobular carcinoma of 0.9 cm (G2). The maximum velocity of 8.40 m/s was measured in the center of the lesion. (b) B-mode and VTIQ images of a 20-year-old woman show a fibroadenoma of 1.4 cm. The maximum velocity of 2.35 m/s was measured in the center of the lesion.

**Figure 2:**
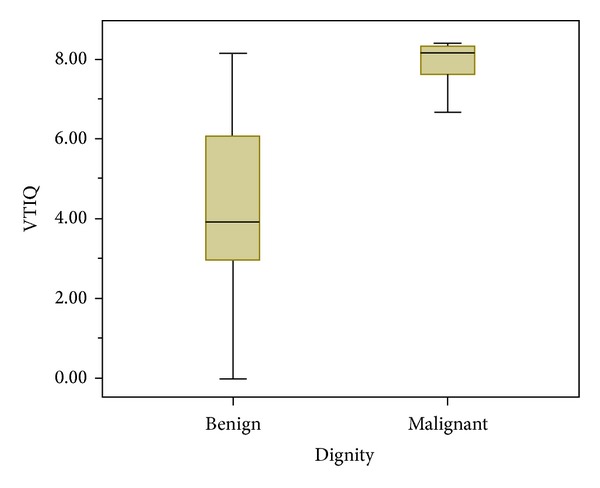
Range of the mean velocities measured in benign (0–8.13 m/s) and malignant lesions (3.06–8.40 m/s).

**Figure 3:**
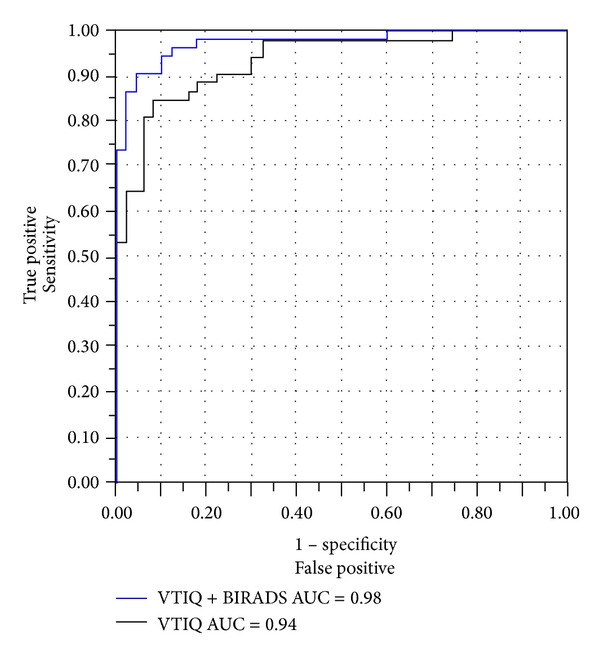
ROC analysis of VTIQ alone and the combination of VTIQ and BI-RADS.

Benign lesions showed a mean shear wave velocity of 4.46 m/s ± 1.87 and malignant lesions of 7.73 m/s ± 1.02 being significantly higher (*P* < 0.0001, Figures 1(a) and 1(b)). The range of the mean velocities measured in benign and malignant lesions is shown in the boxplot (Figure 2). The mean velocity values of both examiners for every BI-RADS category are shown in Table 2. According to the ROC analysis the statistically recommended optimal cut-off, obtained by maximizing the sum of sensitivity and specificity, for VTIQ based on the mean of the five measurements of examiner 1 was 7.13 m/s showing a sensitivity of 85% (46 of 54) and a specificity of 92% (46 of 50). In order to improve the sensitivity the optimal cut-off for clinical decision making concerning a low false negative rate was chosen to be 5.18 m/s. This cut-off yielded a sensitivity of 98% (53 of 54), a specificity of 68% (34 of 50), a positive predictive value (PPV) of 77% (53 of 69), and a negative predictive value (NPV) of 97% (34 of 35). Among the benign lesions 16 of 50 (32%) showed a mean velocity higher than the cut-off of 5.18 m/s, ranging from 5.69 to 8.13 m/s. Within the malignant lesions one out of 54 (1.8%) showed a lower mean velocity of 3.06 m/s. Using standard BI-RADS assessment (with a cut-off level of BI-RADS 4a the indication for biopsy is given) sensitivity, by definition, would be 98% (in our study it was 100%) and specificity 30% (15 BI-RADS 3 cases from a total of 50 benign cases in our cohort), PPV 61% (54 of 89), and NPV 100% (15 of 15). The combination of BI-RADS and VTIQ showed a sensitivity of 98% and a specificity of 82%, a PPV of 86% (53 of 62), and an NPV of 98% (41 of 42) (Table 3). The area under the curve (AUC) for VTIQ alone was 0.94 and for BI-RADS 0.96. The combination of BI-RADS assessment and VTIQ resulted in the best discrimination between benign and malignant lesions (AUC = 0.98; *P* < 0.0001, Figure 3). The bivariate logistic regression analysis confirmed the finding that BI-RADS and VTIQ together improve the prediction of the pathological result of the lesion (*P* value of the model <0.0001).

## 4. Discussion

Up to now the differentiation of breast masses has been based on B-mode ultrasound which suffers from low specificity [5–7]. As a possibility to overcome the low specificity and improve intra- and interexaminer reliability of ultrasound breast diagnostics we evaluated VTIQ as a new elastography method. We investigated the quantitative velocity values of different breast lesions with VTIQ and evaluated its diagnostic performance as a stand-alone method and in combination with standard BI-RADS assessment.

To use elastography on a regular basis in clinical routine it is important that it is highly reliable. The interexaminer measurement agreement for VTIQ had a high positive correlation even between experienced and inexperienced examiners (*r* = 0.93). Also the intraexaminer measurement agreement was consistent.

Two previous studies combining a different quantitative shear wave elastography (SuperSonic Imagine, Aix-en-Provence, France) with B-mode ultrasound showed similar results improving specificity without loss of sensitivity and increasing AUC for the combination of these two methods [10, 11]. For intrarater and interrater reliability prior studies have shown high correlation coefficients for shear wave elastography (intrarater correlation coefficient 0.87 and interrater correlation coefficient 0.87) [19, 20].

To differentiate benign from malignant lesions a cut-off yielding a high sensitivity and specificity is needed.

BI-RADS 3 is defined to be benign in more than 98%. To compare VTIQ with BI-RADS assessment and the combination of these two methods we set the sensitivity of both methods at 98%. For VTIQ this resulted in a cut-off of 5.18 m/s yielding a specificity of 68% compared to 30% for BI-RADS. The combination of VTIQ with BI-RADS yielded a specificity of 82%. It is important to mention that the absolute numbers are not comparable to other studies because they are specific to our study cohort (for example, the specificity for the standard BI-RADS assessment has to be low per definition in our collective, because no BI-RADS 2 and only a few BI-RADS 3 cases were included). But in general it can be stated that VTIQ and the combination of VTIQ and BI-RADS improved the specificity without loss of sensitivity.

Although the sensitivity does not decrease overall, there are single cases where a malignant tumor is soft coded (showing a low velocity) and therefore can be missed. In our collective we had only one case of a ductal invasive cancer which showed a velocity of 3.06 m/s. Regarding the 50 benign lesions 16 showed a higher VTIQ value than the suggested cut-off. Figure 2 shows the range of the VTIQ values for the benign lesions. Due to the very limited numbers of included benign B-mode US BI-RADS cases 4b and 4c outliers strongly influence the mean value resulting in a higher mean VTIQ of B-mode US BI-RADS 4b compared to 4c.

Berg et al. have shown similar results proposing a cut-off of 5.2 m/s for differentiating benign from malignant lesions when adding elastography to BI-RADS assessment [10]. Using another shear wave elastography technique (SuperSonic Imagine, Aix-en-Provence, France) in combination with BI-RADS resulted in an AUC between 0.962 and 0.982 being statistically significantly superior to either BI-RADS or shear wave elastography alone [10, 11, 20]. Combining VTIQ with BI-RADS in our study came to a comparable AUC of 0.98.

It is difficult to objectively quantify the amount of pressure applied while obtaining the VTIQs. Our results show a systematic difference between the measured values of the two examiners indicated by the drift of −0.91 in the orthogonal regression analysis. This could be referred to the different amount of pressure applied by the two examiners. In spite of the difference the test performances—applied by the two different examiners—are the same (AUC (examiner 1) = 0.94 versus AUC (examiner 2) = 0.94).

In the upcoming VTIQ studies the results of a recent study from Barr et. al who dealt with a first semiquantitative approach to measure the precompression could be included [27].

Due to the software version which was available during our study the measurement of the ROI was limited to 8.40 m/s. In the meantime measurements are possible up to 10 m/s. Even if this version would have already been available, the results concerning the cut-off point would be the same. The only difference would have been that the four cancers with 8.40 m/s would have had higher values, the values for the benign tumors would have stayed the same.

For applying VTIQ in the clinical routine we propose scanning and assessing breast lesions with the standard BI-RADS categories. Once BI-RADS assessment has been applied, the velocity of the lesion can additionally be measured with VTIQ. If the measurement is above or below the range of ±1 m/s of the cut-off of 5.18 m/s, one measurement is sufficient and might be used to up- or downgrade the BI-RADS category by one. If the measured value is within this range, the measurement should be repeated to confirm the value. However, this study was limited by the number of cases included. To evaluate the legitimization of up- or downgrading BI-RADS categories by adding VTIQ a larger study with a higher number of cases for each BI-RADS category is needed.

## 5. Conclusion

VTIQ is a highly reliable method concerning intra- and interexaminer agreement. There is a significant difference with respect to the mean maximum velocity of benign and malignant lesions showing malignant lesions to be stiffer. Adding VTIQ to the BI-RADS assessment improves the specificity.

## Figures and Tables

**Table 1:** Absolute and relative frequencies of each BI-RADS category and corresponding mean VTIQ values in m/s.

	Total *n* = 104	Benign *n* = 50	Malignant *n* = 54
BI-RADS			
3	15 (14.4%)	15 (30%)	0 (0%)
4a	32 (30.8%)	29 (58%)	3 (5.6%)
4b	13 (12.5%)	4 (8%)	9 (16.7%)
4c	18 (17.3%)	2 (4%)	16 (29.6%)
5	26 (25%)	0 (0%)	26 (48.1%)
VTIQ Mean Examiner 1	6.16 ± 2.21	4.46 ± 1.87	7.73 ± 1.02
VTIQ Mean Examiner 2	5.71 ± 2.37	3.82 ± 1.79	7.46 ± 1.24

**Table 2:** Mean velocity values for each BI-RADS category in m/s for examiner 1. The values for examiner 2 are shown in parentheses.

	Total *n* = 104	Malignant *n* = 54	Benign *n* = 50
BI-RADS			
3	3.13 ± 1.47 (2.90 ± 1.15)	—	3.13 ± 1.47 (2.90 ± 1.15)
4a	4.89 ± 1.77 (4.01 ± 1.80)	5.93 ± 2.58 (5.26 ± 2.94)	4.78 ± 1.69 (3.88 ± 1.67)
4b	7.33 ± 1.33 (7.25 ± 1.39)	7.71 ± 0.93 (7.75 ± 0.73)	6.47 ± 1.83 (6.13 ± 1.98)
4c	7.61 ± 1.02 (7.37 ± 1.22)	7.83 ± 0.79 (7.63 ± 0.60)	5.81 ± 1.06 (5.24 ± 3.09)
5	7.87 ± 0.77 (7.50 ± 1.24)	7.87 ± 0.77 (7.50 ± 1.24)	—

**Table 3:** Comparison of sensitivity, specificity, PPV and NPV in respect to the proposed clinical cut-off value, standard BI-RADS assessment and the combination of BI-RADS and VTIQ.

	Cut-off 5.18 m/s	BIRADS	Combination VTIQ + BIRADS
Sensitivity	98%	98%	98%
Specificity	68%	30%	82%
PPV	77%	61%	86%
NPV	97%	100%	98%
